# Conductivity Measurement for Non-Magnetic Materials Using Eddy Current Method with a Novel Simplified Model

**DOI:** 10.3390/s25133900

**Published:** 2025-06-23

**Authors:** Changli Yan, Jun Bao, Xuyang Zheng

**Affiliations:** 1 Faculty of Information Engineering and Automation, Kunming University of Science and Technology, Kunming 650500, China; yan.changli@stu.kust.edu.cn; 2 Yunnan Key Laboratory of Intelligent Control and Application, Kunming University of Science and Technology, Kunming 650500, China; 3 Faculty of Civil Aviation and Aeronautics, Kunming University of Science and Technology, Kunming 650500, China; zhengxuyang@stu.kust.edu.cn; 4 Yunnan Dahongshan Pipeline Co., Ltd., Yuxi 653405, China

**Keywords:** eddy current testing (ECT), electrical conductivity, analytical model, phase, excitation frequency, non-destructive testing (NDT)

## Abstract

The eddy current testing (ECT) technique enables efficient and non-destructive conductivity measurement. However, conventional ECT is significantly influenced by the thickness of the material, often resulting in the arbitrary selection of excitation frequency. In addition, complex inverse calculations in the eddy current analytical model pose challenges for practical application. This paper proposes a method for measuring the conductivity of non-ferromagnetic materials based on a simplified analytical model. Firstly, the classical Dodd–Deeds analytical model is simplified based on the electromagnetic properties of materials under high-frequency conditions, resulting in a simplified model that directly relates the coil impedance phase to the material’s conductivity. Furthermore, in combination with a finite element method (FEM) analysis, a frequency selection criterion is proposed, and a corresponding measurement method is developed. This method enables direct conductivity calculation by substituting the measured coil impedance phase into the simplified model. Finally, experiments were conducted to verify the effectiveness of the proposed method. The results demonstrate that the proposed method accurately measures the conductivity of non-ferromagnetic materials over a range of 0.5–58.5 MS/m, achieving absolute and relative errors less than 1.05 MS/m and 1.83%, respectively, without requiring complex inversion calculations or multiple calibrations. This advancement in measurement principles provides a new theoretical foundation and technical pathway for developing online inspection systems and portable instrumentation.

## 1. Introduction

Electrical conductivity is a key indicator for evaluating metallic materials’ electrical performance. It is closely related to the materials’ microstructure, purity, grain size, and internal defects, making it highly significant for quality control and performance evaluation [1,2]. Accurate conductivity measurements not only directly evaluate the conductive performance of metals but also indirectly provide insights into their mechanical properties and thermal conductivity [3]. For instance, conductivity measurements offer a reliable method for verifying the influence of alloy composition during the development of novel alloys, thereby accelerating the material innovation process [4]. In addition, in non-destructive testing (NDT), conductivity measurement plays a crucial role. Measuring the conductivity of metallic components can reveal defects such as internal cracks, porosity, and material degradation [5,6]. With the continuous advancement of industrial applications, the demand for conductivity measurement methods has grown significantly. These methods must not only ensure high accuracy and efficiency, but also demonstrate adaptability to a wide range of material structures and operational conditions. This growing demand highlights the importance of developing robust and versatile measurement techniques based on reliable physical modeling.

The current main methods for measuring the conductivity of metallic materials include the direct current (DC) resistance method [7], the four-point probe method [8], and ECT [9,10]. The DC resistance method is simple but easily affected by contact resistance and temperature variations. Although the four-point probe method effectively reduces the influence of contact resistance on the measurement results, it is suitable only for uniform bulk materials, requires high sample quality, and may cause damage or contamination to the material surface. In contrast, ECT offers significant advantages, including high speed, high precision, non-contact operation, and no need for coupling agents [11]. It is particularly suitable for the NDT and conductivity measurement of conductive materials.

In recent years, numerous scholars have conducted in-depth research on the application of ECT for conductivity measurement. For example, Chen et al. [12] formulated a least squares problem based on the measured and theoretical time-domain induced voltages in a pulsed eddy current field. This approach was used to determine ferromagnetic materials’ conductivity and magnetic permeability. Lin et al. [13] proposed a pulsed eddy current sensor that measures the conductivity of metal plates by analyzing the peak value of the short pulse response. Zhu et al. [14] designed a novel sensor combining inductive and capacitive measurements, where the wire diameter was first inferred from the capacitance value. Then, the conductivity was quickly obtained using a lookup table based on the wire diameter and inductance values. Chen et al. [15] used an LC resonator as the measurement probe and established a logarithmic relationship between the ratio of its resonant frequency and resonant resistance and the material conductivity. Although these studies demonstrate the feasibility and advantages of using ECT for conductivity measurements, most of them rely heavily on an empirical analysis of experimental data trends and lack rigorous theoretical model support. As a result, the generalization capabilities of these methods are often limited when measurement conditions or material properties change. For instance, variations in material thickness can significantly alter the eddy current response, leading to measurement errors and limiting the application of ECT under complex engineering conditions.

Numerous significant methods have been created, as electromagnetic analytical solution models can efficiently, intuitively, and accurately reveal the relationship between measurable parameters and sensor responses. The truncated region eigenfunction expansion (TREE) method [16], created by T. Theodoulidis and J. Bowler, has emerged as a notable analytical methodology. A. Skarlatos et al. [17] extended the TREE method to solve eddy current problems in conductive half-spaces containing vertical cylindrical holes. D. Vasic et al. [18] employed the TREE method to simultaneously measure the electromagnetic properties and inner diameter of conductive pipes. Furthermore, T. Theodoulidis [19] introduced a fast and accurate technique for inverting conductivity profiles from coil impedance measurements. J. Bowler et al. [20] demonstrated the determination of depth-dependent conductivity and permeability via surface potential measurements. Despite the TREE method’s benefits in addressing intricate boundary conditions and multilayer structures, the classical Dodd–Deeds analytical model [21] remains widely used in cases with axisymmetric configurations due to its concise formulation. For example, Xie and Huang et al. [22,23,24] proposed an inverse proportional relationship between the crossover frequency and conductivity. Wang et al. [25] discovered an approximately logarithmic relationship between inductance phase variations and conductivity. Ma et al. [26] demonstrated that the peak frequency of the imaginary part of the inductance variation spectrum is inversely proportional to conductivity. Cao et al. [27] utilized the crossover frequency characteristics of three-frequency eddy current signals to measure the conductivity of metallic films. Although various conductivity measurement methods based on ECT have been proposed, most require multi-frequency or swept-frequency excitation, resulting in high hardware demands. In addition, these methods often involve multiple calibrations, complex integral calculations, and arbitrary frequency selection, all of which reduce detection efficiency.

This paper proposes a conductivity measurement method for non-ferromagnetic materials based on a simplified analytical model to address the aforementioned challenges. Firstly, the classical Dodd–Deeds analytical model is simplified according to the electromagnetic properties of the material under high-frequency measurement conditions. A novel simplified model is then established to describe the direct relationship between the coil impedance phase and conductivity, effectively avoiding the complex integral formulations and parameter inversion required in traditional models. Furthermore, through FEM parametric analysis, the intrinsic relationship between the excitation frequency and material characteristics is revealed. Specifically, when the sample thickness is less than 1.5 mm, the excitation frequency must ensure that the skin depth is no greater than 0.75 times the sample thickness; when the sample thickness is equal to or exceeds 1.5 mm, the excitation frequency needs only to maintain the skin depth of approximately 1.12 mm. This frequency selection criterion enhances the practical applicability of the simplified model. Based on this, a novel three-step measurement method is proposed, comprising “threshold estimation—frequency matching—phase mapping.” First, the conductivity range of the material is estimated using sources such as product manuals. Next, an appropriate excitation frequency is selected according to the frequency selection criterion. Finally, the measured coil impedance phase is substituted into the simplified model to calculate the conductivity directly. Compared with traditional methods, the proposed approach, with its explicit computational model, avoids complex inversion calculations or multiple calibrations. Moreover, it is independent of material conductivity and thickness. The technology facilitates swift and precise conductivity measurements through single-frequency excitation, markedly reducing hardware complexity and enhancing deployment viability. Although this study focuses on homogeneous non-ferromagnetic materials, the generalized potential of the proposed method indicates its promising applicability to more challenging scenarios in the future, such as the in situ industrial inspection of coated or composite structured materials.

## 2. Analytical Derivation of the Measurement Principle

### 2.1. Analysis Model

**Figure 1:**
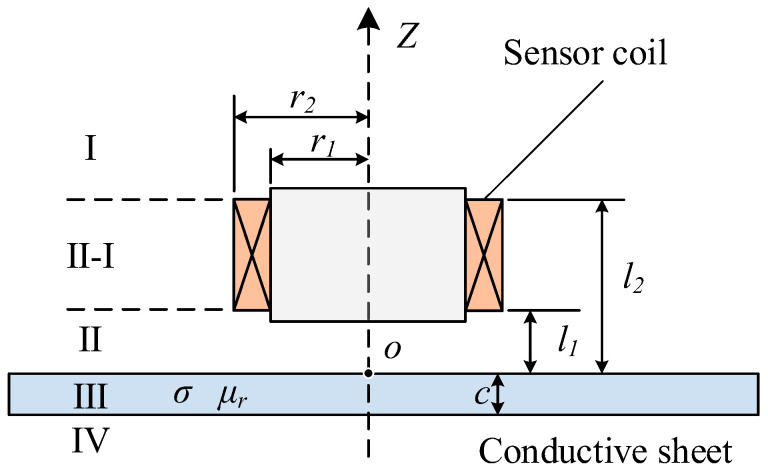
Structure of the EC sensor.

The ECT is an NDT technique based on the principle of electromagnetic induction [28,29]. As illustrated in Figure 1, when a coil carrying an alternating current is placed above a conductive material, eddy currents are induced within the material due to the coupling effect of the alternating magnetic field. The intensity and distribution of these eddy currents are strongly influenced by the material’s electrical conductivity and magnetic permeability. By measuring variations in the coil’s impedance or voltage signals, key electromagnetic parameters of the sample can be inferred.

The impedance of a coil placed on a conductive plate can be derived from the classical Dodd–Deeds analytical model. As shown in Figure 1, the solution domain is divided into four subdomains. The magnetic potential in each subdomain is calculated based on boundary continuity conditions, leading to the final analytical expression for the change in impedance of the eddy current sensor [21,30]:

(1) $$\Delta Z(\omega )=K\int_{0}^{\infty }\frac{Int^{2}(\alpha )}{\alpha^{6}}\cdot {\left(e^{-\alpha l_{2}}-e^{-\alpha l_{1}}\right)}^{2}\cdot \phi (\alpha )d\alpha$$

where

(2) $$K=\frac{j\omega \pi \mu_{0}N^{2}}{\left(r_{2}-r_{1})(l_{2}-l_{1}\right)}$$

(3) $$Int(\alpha )=\int_{\alpha r_{1}}^{\alpha r_{2}}xJ_{1}(x)dx$$

(4) $$\phi (\alpha )=\frac{({\alpha_{1}}^{2}-\alpha^{2})-({\alpha_{1}}^{2}-\alpha^{2})e^{2\alpha_{1}c}}{-{\left(\alpha_{1}-\alpha \right)}^{2}+{\left(\alpha_{1}+\alpha \right)}^{2}e^{2\alpha_{1}c}}$$

(5) $$\alpha_{1}=\sqrt{\alpha^{2}+j\omega \mu_{0}\sigma }$$

where *r*_1_ and *r*_2_ represent the inner and outer radii of the coil, respectively; *l*_1_ and *l*_2_ denote the heights of the bottom and top of the coil; *c* is the thickness of the sample; *σ* is the electrical conductivity; *μ*_0_ is the vacuum permeability; *ω* is the angular frequency of excitation current; *N* is the number of coil turns; and *α* is the integration variable.

Compared to the other integral variables, *ϕ*(*α*) varies slowly to *α*. When *ϕ*(*α*) reaches its maximum, *α* equals the spatial frequency *α*_0_ [31], which is determined by the coil size and lift-off distance. The *α*_0_ can be approximately estimated as the reciprocal of the coil radius. Therefore, *ϕ*(*α*) can be evaluated at *α*_0_ and moved outside the integral:

(6) $$\Delta Z=\phi (\alpha_{0})\cdot K\int_{0}^{\infty }\frac{Int^{2}(\alpha )}{\alpha^{6}}\cdot {\left(e^{-\alpha l_{2}}-e^{-\alpha l_{1}}\right)}^{2}\cdot d\alpha$$

where

(7) $$\phi (\alpha_{0})=\frac{({\alpha_{1}}^{2}-{\alpha_{0}}^{2})-({\alpha_{1}}^{2}-{\alpha_{0}}^{2})e^{2\alpha_{1}c}}{-{\left(\alpha_{1}-\alpha_{0}\right)}^{2}+{\left(\alpha_{1}+\alpha_{0}\right)}^{2}e^{2\alpha_{1}c}}$$

(8) $$\alpha_{1}=\sqrt{{\alpha_{0}}^{2}+j\omega \mu_{0}\sigma }$$

From the above equation, the conductivity variable appears only within the function *ϕ*(α_0_), and the phase of the coil impedance variation depends solely on *ϕ*(α_0_). Therefore, the conductivity can be determined by measuring the phase of the coil impedance variation. The tangent of the phase angle *θ* can be expressed as:

(9) tanθ=Im(ΔZ)Re(ΔZ)=−Re(ϕ(α0))Im(ϕ(α0))

In Equation (7), *ϕ*(*α*_0_) contains two unknown quantities: the conductivity *σ* and the thickness *c*. Since the thickness of the plate is relatively easy to measure, it is assumed that *c* is known. Under this condition, the phase of the coil impedance variation can be used to indicate the material’s conductivity.

### 2.2. Derivation of a Simplified Model for Conductivity Estimation

Due to the difficulty separating the real and imaginary components in Equation (7), Equation (9) is suitable only for the forward calculation of the phase value when both the conductivity and thickness are known. It is impossible to directly derive an explicit relationship between the coil impedance phase and the conductivity. However, according to the study by Lu et al. [32], when the excitation frequency is relatively high, a linear relationship exists between Im (*ϕ*(*α*_0_))/Re (*ϕ*(*α*_0_)) and *α*_0_. When the sample and excitation frequency are fixed, the proportional factor T (*ω*) becomes a constant, which can be derived as follows:

(10) T(ω)=limα0→0Im(ϕ(α0))α0Re(ϕ(α0))

By solving the above equation using MATLAB R2020a symbolic variables and function tools, the following expression is obtained:

(11) Im(ϕ(α0))Re(ϕ(α0))=Re1+j2e(1+j)c2ωμ0σ+1ωμ0σe(1+j)c2ωμ0σ−1⋅α0

Since $e^{jc\sqrt{2\omega \mu_{0}\sigma }}=\cos(c\sqrt{2\omega \mu_{0}\sigma })+j\sin(c\sqrt{2\omega \mu_{0}\sigma })$, substitute it into Equation (11) and further derived:

(12) Im(ϕ(α0))Re(ϕ(α0))=Re((1+j)2(ec2ωμ0σ(cos(c2ωμ0σ)+jsin(c2ωμ0σ))+1)ωμ0σ(ec2ωμ0σ(cos(c2ωμ0σ)+jsin(c2ωμ0σ))−1))⋅α0=2[e2c2ωμ0σ+2ec2ωμσsinc2ωμ0σ−1ωμ0σe2c2ωμ0σ−2ec2ωμσcosc2ωμ0σ+1⋅α0

In addition, when using the ECT to measure the conductivity of materials, the skin depth is typically required to be smaller than the sample thickness [33]. If the skin depth is too large, the electromagnetic field will fully penetrate the material, resulting in distortion of the measurement signal. Therefore, selecting an appropriate excitation frequency based on the sample thickness and the estimated conductivity is essential for practical applications. The formula for calculating the skin depth *δ* is given by:

(13) $$\delta =\sqrt{\frac{2}{\omega \mu_{0}\sigma }}=\sqrt{\frac{1}{\pi f\mu_{0}\sigma }}$$

Equation (13) shows that the skin depth *δ* is inversely proportional to the square root of the excitation frequency *f*, meaning that the skin depth decreases significantly as the excitation frequency increases. According to Equation (13), $c\sqrt{2\omega \mu_{0}\sigma }=2c/\delta$, substituting this relationship into Equation (12) yields:

(14) Im(ϕ(α0))Re(ϕ(α0))=2e4c/δ+2e2c/δsin(2c/δ)−1ωμ0σe4c/δ−2e2c/δcos(2c/δ)−1⋅α0

In Equation (14), $e^{4c/\delta }\approx {\left(54.6\right)}^{c/\delta }$, $2e^{2c/\delta }\approx 2{\left(7.4\right)}^{c/\delta }$, sin(2c/δ) and cos(2c/δ) oscillate within the range of [−1, 1]. Therefore, the following relationship holds:

(15) $$\left\{\begin{array}{c} \left[2e^{2c/\delta }\sin(2c/\delta )-1\right]\;\leq \;\left[2{\left(7.4\right)}^{c/\delta }-1\right]\\ \left[2e^{2c/\delta }\cos(2c/\delta )-1\right]\;\leq \;\left[2{\left(7.4\right)}^{c/\delta }-1\right] \end{array}\right.$$

When the excitation frequency is sufficiently high, especially when it causes the skin depth *δ* to become smaller than the sample thickness *c*, the condition *c*/*δ* > 1 holds, and the following relationship can be established:

(16) $$\left\{\begin{array}{c} e^{4c/\delta }\gg \left[2e^{2c/\delta }\sin(2c/\delta )-1\right]\\ e^{4c/\delta }\gg \left[2e^{2c/\delta }\cos(2c/\delta )-1\right] \end{array}\right.$$

Equation (16) indicates that the contributions of components $2e^{2c/\delta }\sin(2c/\delta )-1$ and $2e^{2c/\delta }\cos(2c/\delta )-1$ in Equation (14) are minimal and can be neglected. Therefore, Equation (14) can be further approximated and simplified as:

(17) Im(ϕ(α0))Re(ϕ(α0))≈2ωμ0σα0=α0πfμ0σ

By substituting Equation (17) into Equation (9), the phase value of the coil impedance variation is derived as:

(18) $$∠\Delta Z=\theta =-\arctan(\frac{\sqrt{\pi f\mu_{0}\sigma }}{\alpha_{0}})$$

By further deriving the phase-simplified model (Equation (18)), a conductivity-simplified model that indicates the relationship between conductivity and the coil impedance phase can be obtained:

(19) $$\sigma =\frac{{\alpha_{0}}^{2}\tan^{2}\theta }{\pi f\mu_{0}}$$

The conductivity-simplified model (Equation (19)) shows that the conductivity of the sample can be directly determined from the measured phase value, resulting in a calculation process that is both simple and efficient.

## 3. Simulation Analysis

### 3.1. Simulation Environment Settings

A two-dimensional axisymmetric FEM was established using COMSOL Multiphysics 6.0. The model simulates the electromagnetic coupling between an eddy current coil and a horizontally positioned metallic sample. This simulation aims to validate the simplified model’s theoretical accuracy through numerical analysis and further explore its applicability and limitations.

**Figure 2:**
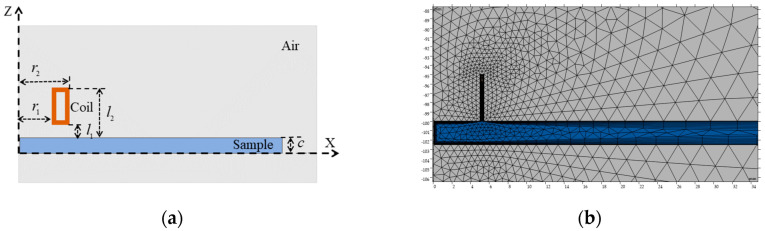
Finite element simulation model: (**a**) Structural schematic. (**b**) Simulation mesh distribution.

Figure 2a shows that the simulated model consists of an excitation coil and the test sample, with the coil’s geometric parameters detailed in Table 1. The magnetic field interface within the AC/DC module was used to solve Maxwell’s equations in the frequency domain. Magnetic insulation was applied as the boundary condition to eliminate edge effects. The mesh density was set to “Extremely Fine,” as shown in Figure 2b, to ensure the reliability of the simulation results. When the sample thickness was set to 0.5 mm, the finite element mesh comprised 62,920 domain elements and 1667 boundary elements, resulting in 151,367 degrees of freedom, with a minimum element quality of 0.034. As the sample thickness increased to 5 mm, the mesh contained 17,647 domain elements and 704 boundary elements, yielding 41,968 degrees of freedom and a minimum element quality of 0.038. All simulations were conducted on a workstation equipped with an Intel Core i9-14900K 3.20 GHz processor and 128 GB of RAM. The average computation time for each parameter set was approximately 9 min.

By comparing the numerical simulation results with the theoretical predictions of the simplified model, the applicable constraint for the excitation frequency was established, providing theoretical and numerical support for engineering applications.

### 3.2. Frequency Selection Criterion

The phase-simplified model (Equation (18)) was analyzed under the coupled effects of excitation frequency, conductivity, and sample thickness to minimize measurement errors. Finite element parametric studies were carried out under the following two scenarios:

#### 3.2.1. Correlation Between Frequency Selection and Conductivity at a Fixed Thickness

**Figure 3:**
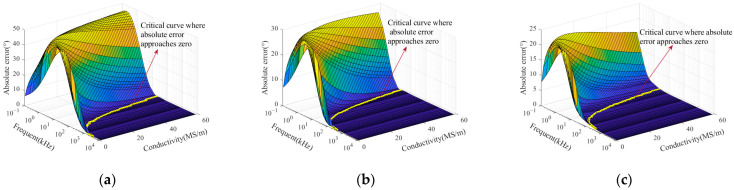
Absolute errors between the simplified model (Equation (18)) and simulation results for different conductivities and frequencies at various sample thicknesses: (**a**) 0.5 mm; (**b**) 2.5 mm; (**c**) 5 mm.

The sample thicknesses in the FEM were fixed at 0.5 mm, 2.5 mm, and 5 mm, covering a typical range from thin sheet materials to conventional industrial components. This setup was used to evaluate the adaptability of the phase-simplified model (Equation (18)) for samples with different thicknesses in practical applications. The conductivity was varied from 0.5 MS/m to 58.5 MS/m in increments of 1 MS/m, and the excitation frequency ranged from 10^−1^ to 10^4^ kHz. After completing the finite element simulations, the phase values of the coil impedance variation were obtained and compared with those calculated using the phase-simplified model (Equation (18)). The absolute error between the two sets of results was calculated, and the error distribution is illustrated in Figure 3.

A critical curve where the absolute error approaches zero can be identified on the surface plot in Figure 3. This yellow curve lies on the conductivity–frequency–absolute error surface and divides it into two distinct regions. On the right side of the curve, the model exhibits convergence, indicating that the curve defines the critical excitation frequency corresponding to different conductivity values. Selected conductivity values and their corresponding critical frequencies described by the curve are listed in Table 2.

**Figure 4:**
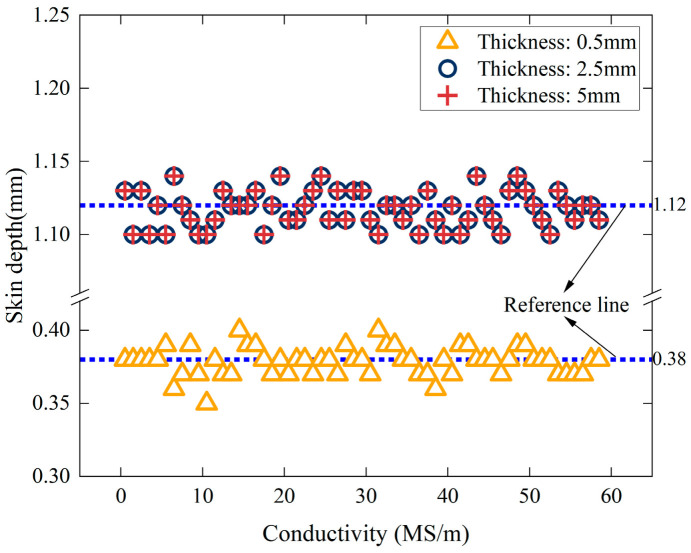
Correlation between skin depth and electrical conductivity under critical conditions.

The error distribution in Figure 3 indicates that when the excitation frequency exceeds the critical threshold, the error of the simplified model approaches zero. Under this condition, the impedance phase of the coil can be directly substituted into Equation (19) to calculate the conductivity. Additionally, Table 2 shows that the critical frequency decreases as the conductivity increases. For sample thicknesses of 2.5 mm and 5 mm, the critical frequencies corresponding to different conductivity values are identical. According to Equation (13), conductivity and excitation frequency are the main factors influencing skin depth. Therefore, the skin depth was calculated for various conductivity values at critical frequencies. The Correlation between skin depth and conductivity under critical conditions is illustrated in Figure 4, with a portion of the calculated results listed in Table 2.

Figure 4 further illustrates that under critical conditions, the skin depth remains constant despite variations in conductivity. However, for the sample with a thickness of 0.5 mm, the skin depth fluctuates between 0.36 mm and 0.4 mm, with an average value of 0.38 mm. For samples with 2.5 mm and 5 mm thicknesses, the skin depth fluctuates between 1.1 and 1.14 mm, with an average value of 1.12 mm. The simulation results are affected by mesh discretization and excitation frequency step size, leading to minor fluctuations in the calculated skin depth.

Therefore, when the absolute error of the simplified model approaches zero, the critical skin depth remains unaffected by conductivity but depends on the sample thickness. Moreover, the critical skin depth stabilizes at approximately 1.12 mm when the thickness exceeds a certain threshold.

#### 3.2.2. Correlation Between Frequency Selection and Thickness at Fixed Conductivity

**Figure 5:**
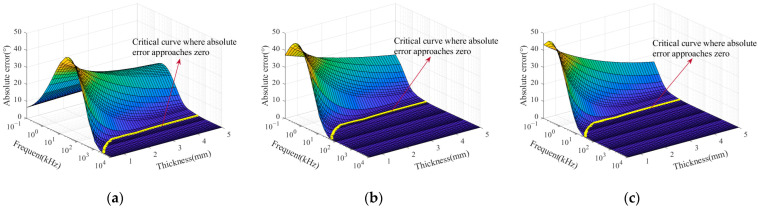
Absolute error between the simplified model (Equation (18)) and simulation results for different frequencies and sample thicknesses at various conductivity values: (**a**) 0.5 MS/m; (**b**) 30 MS/m; (**c**) 58.5 MS/m.

As discussed in the previous subsection, the critical skin depth is correlated with sample thickness. This analysis was conducted by fixing the sample conductivity at 0.5 MS/m, 30 MS/m, and 58.5 MS/m to investigate this relationship, representing typical electromagnetic characteristics of common non-ferromagnetic materials across the full conductivity range. The thickness parameter was varied from 0.5 mm to 5 mm in a step size of 0.1 mm, and the excitation frequency ranged from 10^−1^ to 10^4^ kHz. After the simulations, the coil impedance phase values were extracted and compared with those calculated using the phase-simplified model (Equation (18)). The resulting absolute errors were computed, and the distribution is shown in Figure 5.

A critical curve where the absolute error approaches zero can be identified on the surface plot in Figure 5. This yellow curve lies on the thickness–frequency–absolute error surface and separates it into two distinct regions. On the right side of the curve, the model converges, indicating that the curve defines the critical excitation frequencies corresponding to different sample thicknesses. Selected thickness values and their corresponding critical frequencies are listed in Table 3.

**Figure 6:**
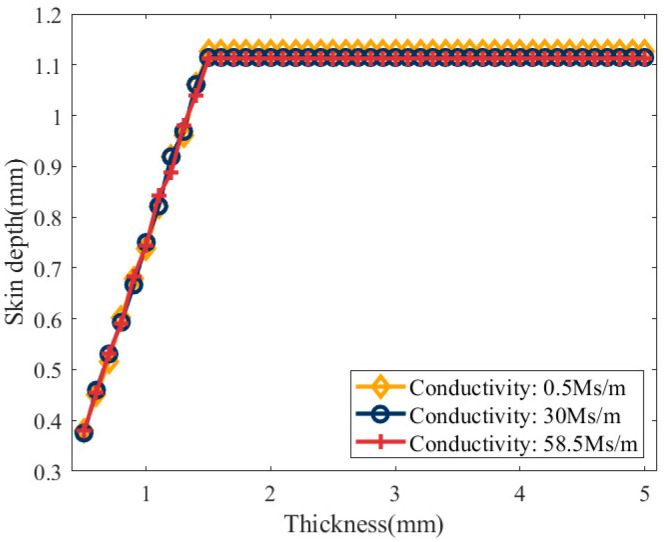
Relationship between skin depth and sample thickness under critical conditions.

The error distribution shown in Figure 5 shows that the critical excitation frequency depends on both conductivity and sample thickness. As thickness increases, the critical frequency initially rises and then gradually stabilizes. Table 3 indicates that when the thickness exceeds 1.5 mm, the critical frequency becomes independent of thickness across different materials. The skin depth under the critical conditions was calculated to further clarify the relationship between the critical frequency and the sample’s thickness and conductivity. The results are illustrated in Figure 6, with a portion of the calculated results listed in Table 3.

**Figure 7:**
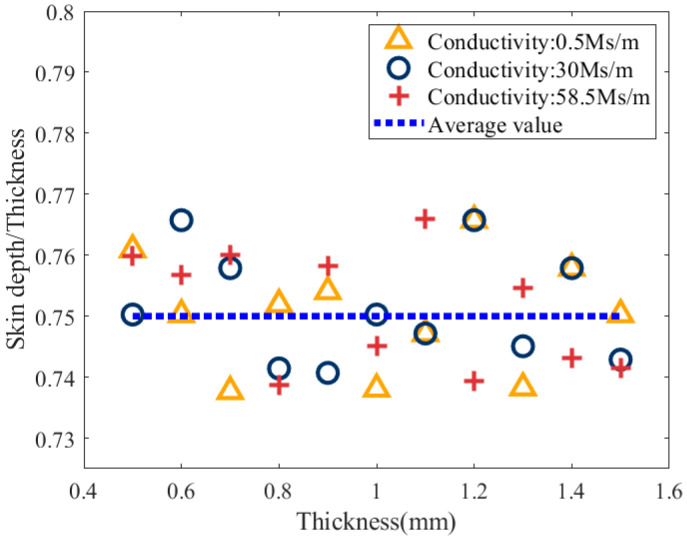
Relationship between the ratio of skin depth to sample thickness and thickness under critical conditions.

Figure 6 further confirms that the critical skin depth is unaffected by electrical conductivity but depends on sample thickness. Specifically, when the sample thickness *c* ≥ 1.5 mm, the critical skin depth converges to approximately 1.12 mm and becomes independent of the sample thickness. When the sample thickness *c* < 1.5 mm, the critical skin depth increases with thickness and exhibits a linear trend. Therefore, for samples with *c* < 1.5 mm, the ratio of the critical skin depth to the sample thickness was calculated, and the results are shown in Figure 7.

Figure 7 shows that when the sample thickness *c* < 1.5 mm, the ratio of the critical skin depth to the sample thickness fluctuates between 0.736 and 0.766, with an average value of 0.75, and remains independent of thickness.

Thus, the excitation frequency can be selected based on the sample thickness and the estimated conductivity. Specifically, for thin samples (*c* < 1.5 mm), the excitation frequency should be chosen to satisfy $\delta =\sqrt{2/(\omega \mu_{0}\sigma )}\leq 0.75c$. For thick samples (*c* ≥ 1.5 mm), it should ensure that δ ≈ 1.2 mm.

Based on the preceding analysis, the excitation frequency selection criterion is derived, as shown in Equation (20), where *σ_min_* represents the minimum estimated conductivity based on product specifications or industry standards. This criterion comprehensively accounts for both sample thickness and conductivity, providing a theoretical basis for frequency selection in practical measurements.

(20) $$f\geq \left\{\begin{array}{c} \frac{8\times 10^{5}}{\pi \mu_{0}\sigma_{\min}},\;\mathrm{if}\;c>1.5\;\mathrm{mm}\\ \frac{16}{9c^{2}\pi \mu_{0}\sigma_{\min}},\;\mathrm{if}\;c\leq 1.5\;\mathrm{mm} \end{array}\right.$$

### 3.3. Conductivity Measurement Method

**Figure 8:**
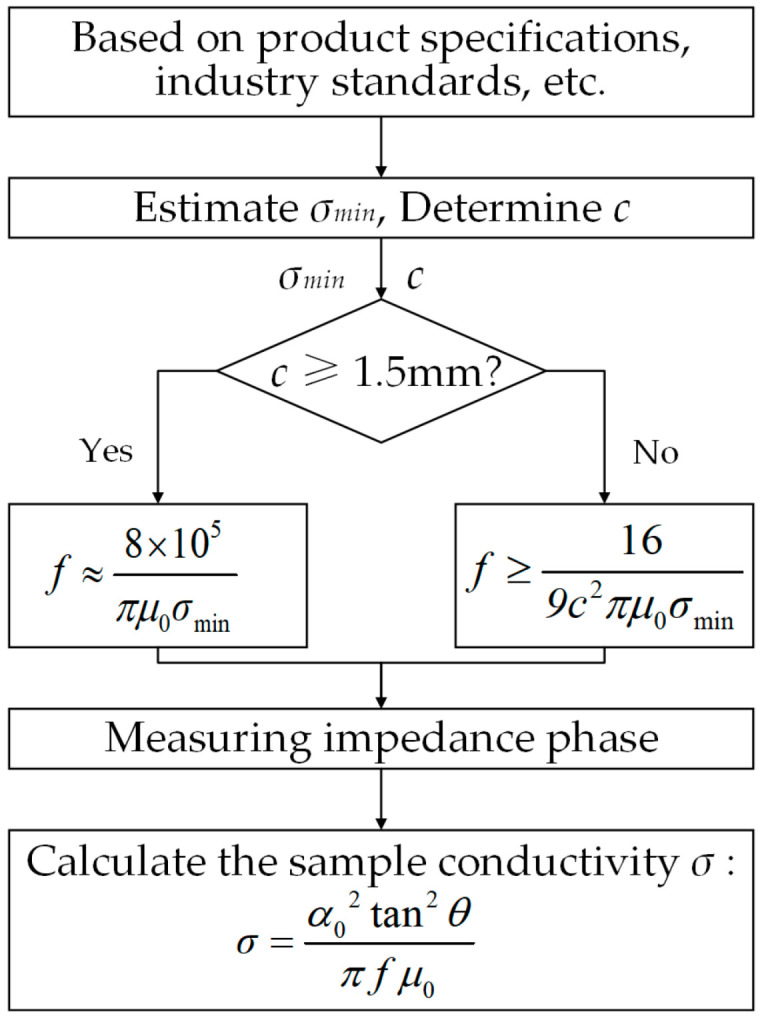
Flowchart of the conductivity measurement method.

Based on the theoretical model and simulation results, a rapid measurement method is proposed for determining the conductivity of non-ferromagnetic materials using the coil’s impedance phase features. By optimizing the excitation frequency selection, the measured impedance phase value can be directly substituted into the simplified model (Equation (19)) to calculate the conductivity. The implementation process is illustrated in Figure 8, and the measurement steps are as follows:

Step 1: Estimate the minimum conductivity *σ_min_* based on product specifications and industry standards, and determine the sample thickness *c*.

Step 2: Select the optimal excitation frequency according to the sample thickness *c* using the frequency selection criterion in Equation (20).

Step 3: Measure the impedance phase of the coil at the selected frequency and directly calculate the conductivity by substituting the measured phase into the simplified model (Equation (19)).

This method integrates optimized excitation frequency selection with a simplified model to enable efficient and accurate conductivity measurements. Compared with traditional swept-frequency ECT methods, it eliminates complex inversion calculations and multiple calibrations, significantly improving detection efficiency. Furthermore, the single-frequency excitation reduces hardware complexity, making the method highly suitable for industrial online detection applications.

## 4. Experimental Verification

**Figure 9:**
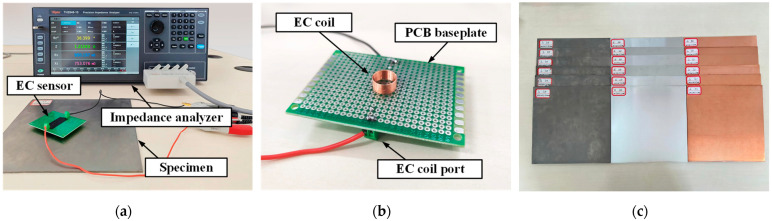
Experimental setup: (**a**) Measurement system. (**b**) EC sensor. (**c**) Test specimens.

An experimental setup was constructed to validate the simplified model and the excitation frequency selection criterion, as shown in Figure 9a. The experimental system comprises test samples, an eddy current coil, and an impedance analyzer (TH2848-10). The eddy current coil, shown in Figure 9b, has the same dimensions as those used in the simulations (Table 1). The coil was placed on the surface of each sample, and the impedance analyzer was used to excite the coil and measure its impedance response. Data acquisition and processing were performed using a computer.

Three representative non-magnetic metal materials were selected for the experiments (Figure 9c): titanium (Ti, TC4, low conductivity, approximately 0.5–0.8 MS/m), aluminum (Al, 1060, medium conductivity, approximately 30–38 MS/m), and copper (Cu, TU1, high conductivity approximately 55–58.5 MS/m). All samples were sized 200 × 200 mm, with thicknesses of 0.8, 1, 1.2, 2, 3 mm, and 4 mm. The sample size ensured that the edge region was significantly larger than the coil area, effectively minimizing edge effects on the measurement results.

The minimum conductivity values of the sample were evaluated according to international standards, and the corresponding excitation frequencies were selected based on Equation (20). Table 4 summarizes the minimum conductivity values and corresponding excitation frequencies for Ti, Al, and Cu.

**Figure 10:**
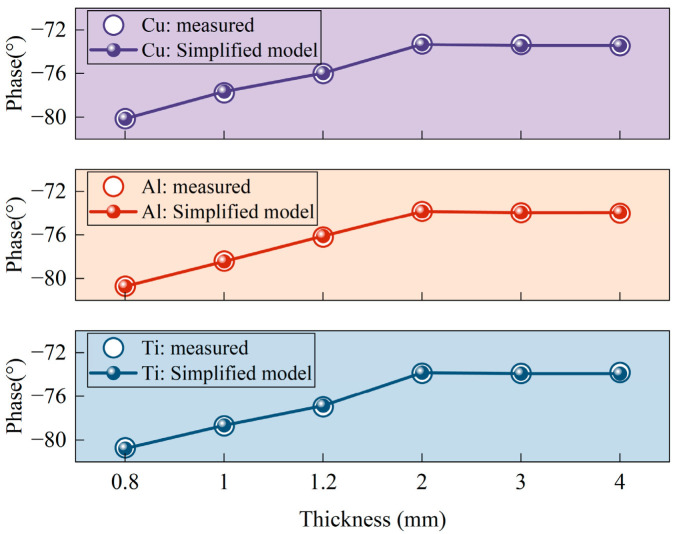
Comparison of measured and calculated impedance phases using the phase-simplified model (Equation (18)) for Ti, Al, and Cu at different sample thicknesses.

Each sample was measured 20 times, and the average value was calculated to ensure data reliability. The measured coil impedance phase values were then compared with those calculated using the phase-simplified model (Equation (18)), as shown in Figure 10.

**Figure 11:**
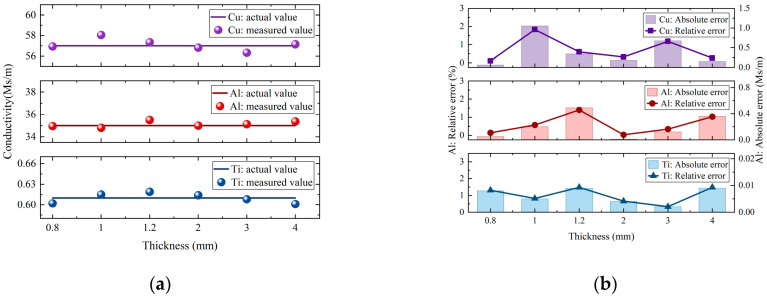
Measurement results and error analysis for copper, aluminum, and titanium samples: (**a**) Comparison between measured and actual conductivity values. (**b**) Absolute and relative errors of the measured results.

Figure 10 shows that the measured coil impedance phase is very close to the impedance phase values calculated using the phase-simplified model (Equation (18)), with only minor deviations. This result verifies the accuracy of the phase-simplified model (Equation (18)) and the validity of the proposed conductivity measurement method. Thus, the measured coil impedance phase values were directly substituted into the conductivity-simplified model (Equation (19)) to calculate the conductivity. The measurement results were then compared with the actual conductivity values. The actual conductivity values were obtained from the product specifications provided by the material manufacturer and used as reference standards to evaluate the measurement error. The final measurement results, including their absolute and relative errors compared to the actual values, are shown in Figure 11 and Table 5.

Figure 11 demonstrates that the measured conductivity values align closely with the actual values, with minimal errors. Importantly, the method’s accuracy is not affected by material conductivity or thickness variations, demonstrating remarkable stability. Furthermore, it avoids complex inversion calculations and multiple calibrations. According to Table 5, the maximum absolute and relative errors are 1.05 MS/m and 1.83%, respectively. The experimental results confirm the effectiveness of both the simplified model and the frequency selection criterion. This study proposes a reliable method for swift and accurate conductivity measurements in non-ferromagnetic materials, providing theoretical support and a technical foundation for developing online detection systems and portable instrument designs.

## 5. Conclusions

This paper proposes a novel simplified model and a frequency selection criterion to address the demand for the conductivity measurement of non-ferromagnetic materials. The method effectively overcomes the challenges of high model complexity and the lack of frequency selection guidance in the traditional ECT conductivity measurement. Firstly, the classical Dodd–Deeds analytical model is theoretically simplified to establish a direct mapping between conductivity and the coil impedance phase, thereby reducing the dimensionality of parameter inversion. Secondly, combined with the FEM analysis, an excitation frequency selection criterion is proposed: for thin specimens (thickness < 1.5 mm), the excitation frequency should ensure that the skin depth does not exceed 0.75 times the sample thickness; for thick specimens (thickness ≥ 1.5 mm), the excitation frequency should ensure a skin depth of approximately 1.12 mm. These guidelines provide clear configuration rules for engineering applications. Finally, a novel three-step measurement method is proposed, which consists of threshold estimation, frequency matching, and phase mapping. This method maintains the hardware simplicity of single-frequency excitation while enabling rapid and wide-range conductivity measurements (0.5–58.5 MS/m). Experimental results demonstrate that the proposed method achieves absolute and relative errors below 1.05 MS/m and 1.83% across the entire measurement range. In contrast to conventional conductivity measuring techniques, the proposed method enables a rapid and precise conductivity estimation under single-frequency excitation, eliminating the necessity for multiple calibrations or complex integral calculations. This considerably decreases hardware complexity and improves the flexibility and viability of engineering deployment. Although this study focuses on non-ferromagnetic materials, the simplified model and proposed measurement method exhibit strong generalizability and scalability. This method may be applied to more intricate situations in the future, including coated or composite structured materials in in situ industrial inspections. These developments will offer a theoretical basis and technical assistance for rapid conductivity assessment across various engineering applications.

## Figures and Tables

**Table 1:** Structural parameters of the EC sensor.

Parameters	Numerical	Parameters	Numerical
$\mathrm{Inner}\;\mathrm{radius}\;\mathrm{of}\;\mathrm{the}\;\mathrm{coil}\;r_{1}$	5 mm	$\mathrm{Lift}-\mathrm{off}\;l_{1}$	0 mm
$\mathrm{Outer}\;\mathrm{radius}\;\mathrm{of}\;\mathrm{the}\;\mathrm{coil}\;r_{2}$	5.4 mm	$\mathrm{Height}\;\mathrm{of}\;\mathrm{the}\;\mathrm{coils}\;l_{2}$	5 mm
Turns of the coil *N*	50		

**Table 2:** Critical excitation frequency and corresponding skin depth for samples with varying conductivity under the condition where absolute error approaches zero.

Conductivity (MS/m)	Thickness: 0.5 mm			Thickness: 2.5 mm			Thickness: 5 mm		
	Frequency (kHz)	$\omega \mu_{0}\sigma$ $\left(10^{6}\right)$	Skin Depth (mm)	Frequency (kHz)	$\omega \mu_{0}\sigma$ $\left(10^{6}\right)$	Skin Depth (mm)	Frequency (kHz)	$\omega \mu_{0}\sigma$ $\left(10^{6}\right)$	Skin Depth (mm)
0.5	3500	13.82	0.38	400	1.58	1.13	400	1.58	1.13
1.5	1200	14.21	0.38	140	1.66	1.10	140	1.66	1.10
2.5	700	13.82	0.38	80	1.58	1.13	80	1.58	1.13
3.5	500	13.82	0.38	60	1.66	1.10	60	1.66	1.10
4.5	400	14.21	0.38	45	1.60	1.12	45	1.60	1.12
︙	︙	︙	︙	︙	︙	︙	︙	︙	︙
54.5	34	14.63	0.37	3.7	1.59	1.12	3.7	1.59	1.12
55.5	33	14.46	0.37	3.7	1.62	1.11	3.7	1.62	1.11
56.5	32	14.28	0.37	3.6	1.61	1.12	3.6	1.61	1.12
57.5	31	14.07	0.38	3.5	1.59	1.12	3.5	1.59	1.12
58.5	30	13.86	0.38	3.5	1.62	1.11	3.5	1.62	1.11

**Table 3:** Critical excitation frequency and corresponding skin depth for samples with different thicknesses under the condition where absolute error approaches zero.

Thickness (mm)	Conductivity: 0.5 MS/m			Conductivity: 30 MS/m			Conductivity: 58.5 MS/m		
	Frequency (kHz)	$\omega \mu_{0}\sigma$ $\left(10^{6}\right)$	Skin Depth (mm)	Frequency (kHz)	$\omega \mu_{0}\sigma$ $\left(10^{6}\right)$	Skin Depth (mm)	Frequency (kHz)	$\omega \mu_{0}\sigma$ $\left(10^{6}\right)$	Skin Depth (mm)
0.5	3500	13.82	0.38	60	14.21	0.38	30	13.86	0.38
0.6	2500	9.87	0.45	40	9.47	0.46	21	9.70	0.45
︙	︙	︙	︙	︙	︙	︙	︙	︙	︙
1.2	600	2.37	0.92	10	2.37	0.92	5.5	2.54	0.89
1.3	550	2.17	0.96	9	2.13	0.97	4.5	2.08	0.98
1.4	450	1.78	1.06	7.5	1.78	1.06	4.0	1.85	1.04
1.5	400	1.58	1.13	6.8	1.61	1.11	3.5	1.62	1.11
1.6	400	1.58	1.13	6.8	1.61	1.11	3.5	1.62	1.11
1.7	400	1.58	1.13	6.8	1.61	1.11	3.5	1.62	1.11
︙	︙	︙	︙	︙	︙	︙	︙	︙	︙
4.9	400	1.58	1.13	6.8	1.61	1.11	3.5	1.62	1.11
5	400	1.58	1.13	6.8	1.61	1.11	3.5	1.62	1.11

**Table 4:** Minimum conductivity and excitation frequency for different sample thicknesses.

Material	$\sigma_{min}\;$(MS/m)	Thickness (mm)	Frequency (kHz)
TC4 Titanium	0.5	0.8	1400
		1	900
		1.2	650
		≥1.5	400
1060 Aluminum	30	0.8	24
		1	15
		1.2	10
		≥1.5	7
TU1 Copper	55	0.8	13
		1	8
		1.2	6
		≥1.5	4

**Table 5:** Measured conductivity and associated absolute and relative errors for samples with different thicknesses.

Material	Thickness (mm)	Actual Value (MS/m)	Measured Value (MS/m)	Absolute Error (MS/m)	Relative Error (%)
TC4 Titanium	0.8	0.61	0.602	0.008	1.31
	1		0.615	0.005	0.82
	1.2		0.619	0.009	1.48
	2		0.614	0.004	0.66
	3		0.608	0.002	0.33
	4		0.601	0.009	1.48
1060 Aluminum	0.8	35	34.95	0.05	0.14
	1		34.80	0.20	0.57
	1.2		35.49	0.49	1.40
	2		34.99	0.01	0.03
	3		35.12	0.12	0.34
	4		35.36	0.36	1.03
TU1 Copper	0.8	57	56.94	0.06	0.11
	1		58.05	1.05	1.83
	1.2		57.34	0.34	0.60
	2		56.82	0.18	0.32
	3		56.33	0.67	1.18
	4		57.15	0.15	0.26

## Data Availability

Data are contained within the article.
